# Social Support-Seeking Behaviours in an Online Support Group for Dental Anxiety: A Content Analysis

**DOI:** 10.7759/cureus.114036

**Published:** 2026-08-05

**Authors:** Mahdy Alshemmari

**Affiliations:** 1 Research and Survey Division, Dental Administration, Ministry of Health, Kuwait City, KWT

**Keywords:** content analysis, dental anxiety, dental fear, online support groups, peer support, social support

## Abstract

Introduction: Dental anxiety (DA) is a prevalent condition associated with dental avoidance and reduced oral health-related quality of life, and online support groups (OSGs) represent an emerging avenue for peer support. This study aimed to assess the types and frequencies of social support sought by individuals with DA within an OSG.

Methods: Internet-mediated research was conducted using qualitative and quantitative content analyses. Publicly available threads from the Dental Fear Central (Oxford, United Kingdom) forum were sampled across designated months in 2022, 2023, and 2024. A final dataset of 271 threads yielding 531 statements was coded and analysed using a modified social support behaviour code framework.

Results: Sharing personal experiences was the most frequent support type (n = 284; 53.4%), followed by seeking informational support (n = 165; 31.1%) and seeking emotional support (n = 52; 9.8%). Encouragement (n = 16; 3.0%), expressions of care (n = 8; 1.5%), expressions of gratitude (n = 3; 0.6%), seeking network support (n = 2; 0.4%), and seeking well-being information (n = 1; 0.2%) were also identified. No statements were coded as seeking esteem support, seeking tangible support, or congratulations.

Conclusion: The current findings can help oral health professionals better understand patient fears and guide the development of tailored digital peer-support interventions. Future research could employ a longitudinal design to explore the long-term impact of OSGs on DA.

## Introduction

Online support groups (OSGs) are internet-based tools that facilitate synchronous and asynchronous interactions among individuals sharing common life experiences [1]. They can be accessed through discussion forums, social media, blogs, and chat rooms [1]. Furthermore, OSGs allow users to communicate across geographical boundaries without financial barriers [1]. Crucially, the inherent anonymity of these spaces fosters uninhibited disclosure [2].

This environment is highly beneficial for individuals seeking information, advice, or emotional validation for sensitive health conditions, including dental anxiety (DA) [2]. DA is a heightened fear of dental treatment affecting approximately 12.4% of adults globally [3]. Individuals with DA frequently delay or avoid dental visits, leading to poorer oral health outcomes and reduced quality of life [4].

Contributing factors for DA include past painful experiences, general anxiety, and lack of social support [5]. Under social support theory, the direct effect and buffering effect hypotheses explain how structured social interactions optimise stress management, whereas deficits in support exacerbate feelings of isolation [6]. OSGs provide a virtual venue to operationalise these supportive interactions [1].

While prior literature confirms that dentally anxious individuals utilise OSGs to articulate fears and seek reassurance [2,7], research applying structured, replicable coding frameworks to evaluate specific typologies of sought support remains sparse. To the author's knowledge, no prior study has applied such a framework specifically within a DA-focused OSG. Understanding the typologies of support sought is clinically relevant, as it can inform which forms of peer support dental professionals signpost patients toward, and can guide the design of digital adjuncts to in-person DA management [2,7]. Accordingly, the aim of this study was to assess the types and frequencies of social support sought within an OSG for DA, using a modified social support behaviour code (SSBC) framework [8], which builds upon SSBC framework by Cutrona and Suhr [9].

## Materials and methods

Study design

This internet-mediated investigation [10] applied a deductive qualitative and quantitative content analysis approach [11] to examine publicly available forum data. Content analysis is a technique used to analyse and make inferences from text and other forms of qualitative information [11]. The deductive component involved mapping forum text onto predefined categories derived from established literature, specifically the modified SSBC [8]. Qualitative content analysis was deployed to interpret the contextual nuance, meanings, and thematic patterns within the forum posts. Quantitative content analysis captured and evaluated the numerical frequency of these elements. This approach was used since it enables analysis of discussion content in its natural context, avoids biases associated with participant interaction, and captures data from geographically dispersed populations [10].

Online support group selection

To identify relevant OSGs, a systematic Google (Alphabet Inc., Mountain View, United States) search was executed using target keywords: "anxiety, bulletin boards, community, dental, fear, forum, group, online, peer, support, website". OSGs were included if they exclusively addressed DA and were publicly accessible without registration; OSGs were excluded if they were non-English, addressed conditions other than DA, or required registration to read content. Eight OSGs were identified; six (Dentistry Forums, Health Boards, Mental Health Forum, Phobia Support Forum, Reddit (Reddit, Inc., San Francisco, United States), and Scope (Scope, London, United Kingdom)) were excluded because they addressed conditions unrelated to DA or provided only professional support, and one (Facebook, Meta Platforms, Inc., Menlo Park, United States) required registration. One OSG met all criteria: Dental Fear Central (Oxford, United Kingdom) [12].

Thread selection

Threads were included if they related to DA, contained at least one support-seeking statement according to the SSBC framework, were written in English, and were posted in even months of 2022 and 2023, plus February, April, and June 2024. This method was used since it ensured a manageable dataset within a defined timeframe while reducing the influence of subjective judgment in thread selection [10]. Threads were excluded if unrelated to DA or outside the defined timeframe. Twenty-five threads were randomly sampled from each of the 15 qualifying months using an electronic random number generator [10], yielding a sampling pool of 375 threads. These threads were then screened for the presence of at least one SSBC-codable statement.

Data analysis

Coding was executed via a modified SSBC framework [8], which classifies social support-seeking behaviour into 11 types: seeking informational support, seeking well-being information, seeking emotional support, seeking esteem support, seeking network support, seeking tangible support, sharing personal experiences, expressions of gratitude, congratulations, expressions of care, and encouragement.

This modified SSBC builds on SSBC framework by Cutrona and Suhr [9], which identified five support categories (informational, emotional, esteem, network, and tangible), incorporating subsequent additions by Coursaris and Liu [13] such as sharing personal experiences, gratitude, and congratulations. Further refinements were introduced by Algtewi et al. [8], including categories for seeking well-being information, expressions of care, and encouragement. Each thread was reviewed and coded multiple times; multiple codes were assigned where a statement reflected more than one support type. Coding was conducted manually using a colour-coding system, whereby each of the 11 support types was assigned a distinct colour and corresponding statements within the threads were highlighted accordingly.

Ethical considerations

This study analysed publicly available, anonymised online data and did not involve direct participant interaction or collection of identifying information. No ethical approval was required. The research was conducted in accordance with the British Psychological Society guidelines for internet-mediated research [14].

## Results

Main findings

Of the 375 randomly sampled threads, 104 did not contain any statement corresponding to a category within the modified SSBC framework and were excluded. The remaining 271 threads, yielding 531 statements, were analysed. Most threads (n = 159; 58.7%) contained two types of social support, (n = 75; 27.7%) contained one type, (n = 34; 12.5%) three types, and (n = 3; 1.1%) four types. No threads contained more than four types (Table 1).

**Table 1:** Distribution of social support types sought per thread

Number of support-seeking types per thread	Threads (n)	%
One type	75	27.7
Two types	159	58.7
Three types	34	12.5
Four types	3	1.1
More than four types	0	0
Total	271	100

Of the 531 statements, sharing personal experiences was the most frequent type (n = 284; 53.4%), followed by seeking informational support (n = 165; 31.1%) and seeking emotional support (n = 52; 9.8%). Encouragement (n = 16; 3.0%), expressions of care (n = 8; 1.5%), expressions of gratitude (n = 3; 0.6%), seeking network support (n = 2; 0.4%), and seeking well-being information (n = 1; 0.2%) were also identified. No statements were coded as seeking esteem support, seeking tangible support, or congratulations (Table 2).

**Table 2:** Types and frequencies of social support sought across all statements

Type of social support sought	Statements (n)	%
Seeking informational support	165	31.1
Seeking well-being information	1	0.2
Seeking emotional support	52	9.8
Seeking esteem support	0	0
Seeking network support	2	0.4
Seeking tangible support	0	0
Sharing personal experiences	284	53.4
Expression of gratitude	3	0.6
Congratulations	0	0
Expression of care	8	1.5
Encouragement	16	3
Total	531	100

Sharing personal experiences

Sharing personal experiences was the most frequently identified support type (n = 284; 53.4%). Posts included narratives of procedural anxiety, accounts of past dental trauma, descriptions of how anxiety affected daily life including sleep, and reflections on prolonged dental avoidance. Most messages focused on panic attack stories.

For instance, one member who experienced panic over fillings posted: "I need three fillings, and apparently, they have to be silver. The dentist offered to do them immediately, so I signed the forms, got ready, and sat down. However, I had a severe panic attack, fainted three times, and needed oxygen for about 15 minutes."

Another example of panic involved someone's fear of injections: "I went for a routine check-up, and the dentist, looking troubled, informed me I had cavities in two back molars that needed fillings and numbing. As someone with a lifelong fear of needles, I panicked, jumped out of the chair, and frantically cried "No! No! No!" while looking at the nurse, the dentist, and my mum."

A further example included a member who panicked over the sensation of getting numb: "I'm not afraid of the needle or the drill itself, but I fear the sensation of the injection. It makes me feel like I can't breathe and that my throat might close up. This fear causes me to panic, cry, and feel like I'm making a complete fool of myself."

Seeking informational support

Seeking informational support was the second most frequent type (n = 165; 31.1%). Statements comprised specific questions aimed at obtaining factual information, advice, or assistance in evaluating a situation. Procedure-related queries predominated.

For instance, many members expressed anxiety about injections. One person posted: "Is anyone else terrified of anesthesia and its effects? How do you manage to relax? I'm extremely anxious and wondering if they numb the roof of your mouth for an upper tooth procedure, does anyone have any information?"

Individual-related questions often focused on pain management. For example, one member asked: "This tooth frequently causes infections and abscesses, leading to intense pain. My anxiety has been so severe that I couldn't visit the dentist for sedation. Now, I'm struggling to sleep due to the pain, is this normal, and should I continue waiting for my medication to take effect?"

Provider-related concerns were less frequent and focused on issues with dentist behaviour. One person posted: "I have a strong fear of dentists and their staff acting in ways that are pushy, aggressive, deceptive, angry, raise their voices, use high-pressure tactics, or are only focused on making money. This fear makes it very difficult for me to communicate with them, ask questions, or negotiate. I would really appreciate any stories, tips, or advice."

Seeking emotional support

Seeking emotional support was the third most frequent type (n = 52; 9.8%). Posts conveyed feelings of anxiety, distress, or helplessness and sought comfort or reassurance. Most posts addressed how dental issues were affecting overall well-being.

For example, one member shared: "Mainly my goal is to be happy and healthy, and my teeth and mouth are really bringing me down in every aspect of my life. I'd appreciate any support or words of encouragement with this from anyone."

Another member, whose DA was impacting their daily life, posted: "Does anyone know where people can have therapy to talk about their dental problems and phobias of dentists and talk about other things that they are worried about? It is taking over my life and I am in tears every day and I really need to talk to someone."

Another instance involved an individual preparing for their first dental appointment in 15 years. They posted: "Just looking for support, I have my first dentist appointment in 15 years coming up. I have already told the dentist I'm scared. I have a real phobia of the dentist due to past dental trauma. I'm terrified and extremely anxious."

Other support types

Encouragement (n = 16; 3.0%) included messages expressing hope and confidence. One example involved a member who encouraged themselves by recalling a past positive experience with nitrous oxide: "I'm hoping everything will be fine, and I keep reminding myself how relaxed I was about needles while on nitrous. I even opened my mouth willingly with no hesitation! I know I can do it again!"

Expressions of care (n = 8; 1.5%) focused on conveying concern for others. One example involved a user who expressed concern for others whilst sharing their own struggles with anxiety: "The anxiety is consuming all my thoughts, and I just needed to express it instead of keeping it bottled up. I apologise to anyone else going through this."

Expressions of gratitude (n = 3; 0.6%) included messages expressing gratitude for previous support received from the community. One member posted: "Thank you for helping me express my anxiety. I'm glad to say that it's now at a healthy level."

Seeking network support (n = 2; 0.4%) comprised statements requesting contact with others in a similar situation. An individual who was anxious about the possibility of needing a tooth extraction wrote: "I'm really scared to make the call and hear that I need a tooth pulled. I'm not sure what to do and would really like to talk to someone."

Seeking well-being information (n = 1; 0.2%) involved a single statement directed at supporting a third party rather than the poster. One member expressed concern for a family member with neglected dental health and asked: "I'm posting this mainly out of concern for my father. I'm unsure if he's afraid or simply doesn't think it's necessary, but he definitely needs to visit the dentist and address his dental issues. What should I do now? I want him to get treated and stay healthy."

Absent support types

No statements were coded as seeking esteem support, seeking tangible support, or congratulations.

## Discussion

This study classified the types and frequencies of social support sought by individuals with DA within an OSG using a validated content analysis framework. Sharing personal experiences and seeking informational support together accounted for 449 (84.5%) coded statements, suggesting that these were the dominant functions of the DA-specific online forum. This pattern is consistent with OSG research across other health conditions that employed the SSBC framework, such as head and neck cancer [8], eating disorders [15], Essure-related complaints [16], and skin cancer [17], where sharing personal experiences and seeking informational support have been identified as primary functions.

In contrast to the pattern found in the current study, other studies across different health conditions that employed the SSBC framework, such as transgender issues [18], HIV/AIDS [13], and diabetes mellitus [19], have found the opposite. The high frequency of sharing personal experiences is consistent with the buffering effect theory of social support [6]. This theory proposes that the perceived availability of support encourages individuals to disclose their experiences, which reinforces a protective buffer against stressors and strengthens coping abilities.

Although both support types were frequently identified, sharing personal experiences was more prevalent than seeking informational support. This pattern aligns with several OSG studies [8,16,18] but contrasts with others where informational support ranked first [13,19]. The prominence of seeking informational support is similarly consistent with the direct effect theory [6]. Acquiring knowledge may directly enhance well-being by promoting informed decision-making and increasing individuals' sense of control over their DA.

Beyond the two dominant types, emotional support was the third most frequent type (n = 52; 9.8%). This may reflect the supportive environment of a DA-specific forum, where members feel safe to express psychological vulnerability, consistent with OSG research on head and neck cancer [8], HIV/AIDS [13], and transgender issues [18]. The complete absence of esteem support, tangible support, and congratulations is consistent with the nature of an online community addressing a fear-based condition [2,7]. The absence of tangible support has been similarly reported in Essure-related complaint forums [16]. Moreover, the absence of esteem support differs from findings in head and neck cancer [8] and HIV/AIDS communities [13], suggesting that performance validation may be more relevant to conditions involving greater social comparison or stigma.

Limitations

Several limitations should be acknowledged. As data were drawn from a single OSG, the current findings may not generalise to users of other platforms such as Reddit. The restriction of inclusion to English-language threads may further limit the generalisability of the findings to non-English-speaking populations with DA. Moreover, the sample size of 271 threads may not represent the full diversity of individuals with DA. Coding was conducted by a single researcher; the absence of a second coder or a formal inter-rater reliability assessment may have introduced an element of subjectivity into the categorisation process. Furthermore, internet-mediated research cannot capture non-verbal cues [10]. Additionally, the absence of demographic data precludes subgroup analysis [20]. Most importantly, content analysis and the use of predetermined categories do not capture the depth of individual experience [11].

## Conclusions

The current findings map the social support landscape of DA-specific online forums. The findings can inform the development of evidence-based digital peer-support platforms and empower oral health professionals to better understand, anticipate, and manage the online behaviours and underlying anxieties of their patients. Future research could employ a longitudinal study design to explore the long-term impact of OSGs on DA, including changes in anxiety levels, oral health-related quality of life, and support-seeking behaviours.
